# "The Hospital" Nursing Mirror

**Published:** 1900-09-22

**Authors:** 


### "The Hospital" Nursing Mirror.

Being the Nursing Section of "The Hospital."

[Contributions for this Section of "The Hospital" should be addressed to the Editor, The Hospital, 28 & 29, Southampton Street, Strand, London, W.C., and should have the word "Nursing" plainly written in left-hand top corner of the envelope.]

### Notes on News from the Nursing World.

### MALE NURSES AT ALDERSHOT.

It has been stated in some of the papers that "the experiment is being tried at Aldershot of replacing the trained hospital orderlies by properly-trained civilian male nurses," and about 25 are said to have been thus engaged for duty in the Cambridge and Connaught Hospitals. The fact is, however, that a certain number of male nurses have been given a temporary engagement only of six months to replace the men of the R.A.M.C. at present in South Africa. These male nurses are not in lieu of the sisters of the Army Nursing Service Reserve. The latter are largely employed in military hospitals in the United Kingdom at present.

### NURSING KAFFIRS AT MARITZBURG.

An Alexandra Nurse writes us under date of August 24th: "Since my return from sick leave I have resumed nursing on the old lines, with this difference, that now my patients are greatly diminished. Only some thirty each morning seems very few indeed, after the larger numbers formerly dealt with. But it gives me more time to massage their stiffened joints and shoulders, &c. So many are victims to rheumatism. They are still very grateful for attention shown them, all the more, that being outdoor patients they do not expect to be so carefully treated. Several are suffering from the effects of pleurisy and pneumonia, and this week I have had quite a rush of sprained knees or ankles to deal with. Frequently, I have also a few Kaffirs attending. These are generally accident cases. Being employed as drivers or in the Cavalry yard they are sometimes kicked or bitten by the horses. They have a very curious custom, if suffering from a pain in the side or back, of having the part cut or scored with a sharp knife to let the bad blood out. But I think they prefer my treatment, and consider me rather more merciful. Great is their joy and sense of superiority when they are dressed with nice white wool and clean bandages just like Tommy."

### A REMINISCENCE OF SIR WILLIAM STOKES.

The same correspondent sends the following reminiscence of Sir William Stokes: "This week we have had the very sad duty to perform of laying the late Sir William Stokes in his grave. Courteous and genial, he was greatly liked by all. I had the gratification of meeting him, and he asked if I came from Edinburgh. On my replying 'No; I belong to Glasgow,' he said quite joyously, 'Oh, I like Glasgow; I got my wife from there.' It was a simple and human little speech that quite won my heart. During the last two weeks of his life Sir William came into the inspection-room where I was attending the sick, and paid me a visit for a minute or two. On the last occasion, as I was dressing the head of a Cape boy who had been thrown while driving an ambulance waggon, he said 'That is right, we can't do without them just now'; and so passed out, leaving a pleasant memory behind."

### NURSES FROM THE TRANSVAAL.

A number of Russian and Dutch nurses, for whom there is no further need in the Transvaal, were landed at Flushing on Thursday. They returned, under the auspices of the British Consul, from South Africa in the "Braemar Castle," and on their arrival they produced, with much exultation, a letter from Lord Roberts thanking them for their kindness to wounded British prisoners. One of them, at any rate, had some work on the way, for a German woman gave birth to a son on board, who is to be christened "Julius Braemar."

### THE NURSING AT ESTCOURT.

With reference to the statement made by one of the Sisters in charge of the "Oratava" in our issue of September 8th, that the Superior of the Convent at Estcourt died of starvation, because she had given up her food to the soldiers, and not, as was supposed, from enteric fever, we are informed by a correspondent who was acting as orderly in the hospital from November, 1899, to the following April, that "during all that time the hospital was never short of food, and all patients, both in the hospital and sanatorium, had all they could wish for or were allowed." He further declares that "the Superior died of internal inflammation caused by cold and damp contracted in her labours for the welfare of the soldiers." In making this assertion, he says he is only anxious to correct what he considers "a slur upon the Convent at Estcourt, the military authorities, the civil doctors, and all concerned," and "not to throw a slight upon the work done by the Rev. Mother," of whom, he adds, "Nothing could exceed the nobleness, devotion, and patience which she displayed in her care and attention to the inmates of the hospital."

### ONE YEAR'S "TRAINING."

It is suggested in a Jewish paper that a number of Jewish girls should be trained as nurses in Jewish wards in London hospitals. The suggestion is prompted by a visit on the part of the author to a Jewish hospital in one of the larger cities of the Rhine, where, he states, pupils between twenty and thirty are trained "in one year to fully satisfy the much-increased requirements of the present day." But there exists no kind of barrier to the admission of Jewish young women as probationers to any of our great hospitals; and, as a matter of fact, there are Jewish nurses in some of them, only there can be no distinction between their training and that of other probationers. Whatever may be the case in a city on the Rhine, one year is certainly not a sufficient time to satisfy the requirements of the present day in England, and it is quite useless to proceed with any movement which rests upon such a misconception of the situation.

### THE MEMBER FOR THE STRAND AND NURSING AT MORETONHAMPSTEAD.

One of the many benefits which Mr. W. F. D. Smith, M.P., has conferred on the Moretonhampstead district since he has had a stake in the county has been the support he has given, in conjunction with his wife, Lady Esther, to the nursing movement. At the present time he is building, at his own expense, a cottage hospital which is to cost £1,500, and which, it is hoped, will be opened early next year. Mr. Smith is president of the Moretonhampstead District Nursing Association, and at the annual meeting last week he stated that the management of the new hospital would be in the hands of a representative committee. He proposed himself to appoint the first committee, and subsequently the power will devolve upon the subscribers. As it will not be possible for the district nurse to reside at the hospital, the nursing association will continue to be managed by a separate committee.

### A NEW TRAINING SCHOOL.

The Guardians of the Kingston Union, Surrey, have decided to make their new infirmary a training school for nurses. A resolution was passed at their last Board meeting "That application be made to the Local Government Board for permission to separate the infirmary from the workhouse, to appoint a resident medical man, and to train nurses at the infirmary."

### THE NEWRY GUARDIANS AND THE LOCAL GOVERNMENT BOARD.

The Newry Board of Guardians are very indignant with the Irish Local Government Board because the latter will not relax their regulations in order to allow the Guardians to employ a nurse who is not fully qualified. It appears that the nurse was the midwife of one of the dispensary districts of the Monaghan Union, and was appointed night nurse at the Newry Workhouse Infirmary by the Guardians, on the strength of two testimonials. But when these were sent to the Local Government Board for the purpose of obtaining the requisite sanction, the reply elicited was to the effect that "nothing short of a certificate from a medical and surgical hospital could be regarded as satisfactory evidence of the lady's qualifications as a nurse." The Board, therefore, declined to approve the appointment, and requested that the Guardians would proceed to a fresh election. This the Guardians have, so far, refused to do, but they will find that in the end they must submit. If they had consulted the Irish Local Government Board before they made their choice, or had paid any regard to the regulations with which they must or should be familiar, they would have avoided placing themselves and the nurse, whose qualifications do not come up to the required standard, in an awkward position.

### NURSING ASSOCIATIONS IN CLEVELAND.

It is pleasant to learn that the value of trained nurses is well known in some parts of Cleveland, and that many of the miners, who form the bulk of the population, have paid a high tribute to their skill and efficiency. In the Skelton and Guisbro' district there is already a nursing association at work; it is proposed to form one at Brotton, and at Skinner's Grove a start has just been made. A number of influential ladies, with the Marchioness of Zetland at their head, have interested themselves in the project, and a committee has been appointed. With regard to subscriptions, Lord and Lady Zetland, Mr. A. E. Pease, M.P., Pease (Limited), and the Skinner's Grove Iron Company have promised liberal contributions; and what is, perhaps, better still, the workmen employed by the two firms have also agreed to subscribe a penny a month each. The rules of the association provide that subscribers who require the services of the nurse shall be entitled to them free of charge, but a nominal fee will be imposed in the case of non-subscribers. It is hoped that the nurse herself will shortly take up work in the locality.

### MEMORIAL TO THE FOUNDER OF THE DARLINGTON NURSES' HOME.

With a view to perpetuating the memory of Mr. Arthur Pease, who represented Darlington in Parliament, and was the founder of the local Queen's Nurses' Association, a tablet has been inserted on the front of the home. The unveiling ceremony will take place shortly. The inscription upon the tablet is as follows: "The Arthur Pease Memorial Home for Queen's Nurses. November, 1899. Contributed to by a large number of subscribers, who thereby seek to perpetuate his memory. Arthur Pease, born 12th September, 1837, died 27th August, 1898. A member of the Corporation of Darlington, 1837 to 1898; Member of Parliament for the Borough of Whitby, 1880 to 1885; Member of Parliament for the Borough of Darlington, 1895 to 1898."

### A POPULAR NOVELIST AND A NURSING ASSOCIATION.

Mr. Stanley Weyman, in his capacity of a subscriber to the Ruthin Nursing Association, has exercised his influence in favour of the retention of two nurses. It was proposed, in consequence of the small contributions to the association, to dispense with the services of one, but at the annual meeting the Mayor of Ruthin and the Rev. Chancellor Bulkeley Jones, as well as Mr. and Mrs. Stanley Weyman, urged that a great effort should be made to raise the amount necessary to pay the salaries of two. The Mayor, who is a medical man, said that to do away with one nurse would be a great calamity. He insisted that a district nurse and a midwifery nurse were equally wanted, and he added that he should do all in his power to fight against any attempt to combine both classes of work. Happily, there is no immediate fear of such an attempt being made. The proposal that because there is only one nurse at Denbigh, Corwen, and Llangollen, there should be only one at Ruthin, was, in the end, unanimously set aside.

### THE CONDITIONS OF NURSING IN PARIS.

A well-informed correspondent in Paris, who is herself a nurse of wide experience, sends us the following:—

"Many nurses say they would like to come to Paris, but before doing so I should advise them to consider it well and know what to expect. A nurse who comes alone and on her own responsibility may think herself lucky if she keeps herself for the first two years. There are many private nurses, some living alone, others with friends, and a few homes where several live together, receive their own fees, and share qually in the rent and other expenses, taking cases in turn.

Then there is the Hollond Institute, where I believe the nurses are paid by the year. Many nurses passing through with patients are tempted to remain by the high fees we receive, and also by the doctor telling them that there is plenty of work, and that he will be able to employ them. But he does not always do so. The doctors, as a rule, are very polite and kind to English nurses, and very glad of their services. There are many good English doctors here, and their practice is mostly in hotels and among English and American visitors. Now English trained nurses are becoming known in France, and are getting more work in French families. A nurse coming to Paris will do well if she joins other nurses already established, but I should not advise her to come alone. It is hard and uphill work. The expenses of living are very great for a foreigner. It is also quite possible to be out of employment for several months at a time. From March till July there is plenty of work, then Paris is empty, and there is nothing doing. Financially speaking, we do not like English patients, for they generally tell us they can get the best nurses in England for £1 a week, and consider our terms terrible, but personally we like them, as they are more considerate to nurses, and do not expect day and night duty, neither do they treat us as inferiors. When a nurse comes she generally works for English doctors only. Gradually she gets to know French ones, and thus begins to work among French families. If she happens to please a good French doctor, she need have no fear of lack of work; if he speaks English he likes to improve it, and he is very often astonished when he finds out how an English nurse is trained. Here, however, I must remark that 'tact' is just as necessary to her as 'training,' and she need not be at all surprised if, after she has nursed two or three cases for him, she finds he imagines he has trained her. He will always treat her with courtesy before her patients, which is more than he does his French nurse. The first time I nursed for a French doctor he used to give his orders all round, to the patient, the mother, the maid, the governess, and in fact, to everybody but the nurse. He does not do so now; he gives them to me."

### A SUCCESSFUL SALE OF WORK.

No less than £319 has been realised by a two days' sale of work at Garstang in aid of the District Nursing Association. This result is highly creditable to the promoters of the movement, and it also shows that the public of Garstang are willing, indirectly, to render support to the work. It is a pity, all the same, that in order to keep the association going it should be requisite to organise a bazaar. The report for the last year shows that 149 cases were attended, and 1,785 visits paid, and two nurses are obviously wanted. But so far the subscriptions have not sufficed to provide the salary for a second nurse, and Miss Dell has discharged her duties single-handed. We hope that in future the residents, of all classes, will recognise that it is their duty, and must be to their advantage, to put the association on a sound basis, instead of permitting it to depend for funds upon a special effort.

### A YOUNG MOTHER'S MESSAGE TO SOUTH AFRICA.

"A LITTLE incident that came under my notice," writes a correspondent, "perhaps would interest other nurses. B——, formerly in the Hussars, but for some time on the Reserve, was engaged to be married when the war broke out. He thought himself, however, pretty safe not to be called up, and was married on a certain Thursday. Alas! on the following day the summons came to rejoin. As war is inexorable, the adieux had to be said, and the pretty little new home shut up. The young bride went to his mother to pass the time of his absence. As the months went by she found she had to busy herself in making certain tiny garments, and in July a baby daughter came to gladden her. I was shown a charming photograph of the grandmama, the young, girlish-looking mother, and the bonny, wee baby holding in its tiny hands its father's good conduct medal (received in former service). 'I reckon this photo is a "message" as will make Joe proud,' said his mother, and I thought so too. The little touch of sentiment in the medal one would have scarcely looked for with homely folks!"

### HANAN MEMORIAL FUND.

MAJOR BALLANTINE sends us the final list of subscribers to the Hanan Memorial Fund:—

| | | | | | | | |
|---|---|---|---|---|---|---|---|
| Nurse Shankland ... | £0 | 10 | 0 | Nurse Porter ... | 0 | 5 | 0 |
| Nurse Heriot ... | 0 | 5 | 0 | Nurse Ventris ... | 0 | 5 | 0 |
| Nurse Hall... ... | 0 | 2 | 0 | Nurse Sandiford ... | 0 | 5 | 0 |
| Mrs. Thomas (*neé* Foulis) ... ... | 1 | 1 | 0 | Nurse Roberts ... | 0 | 2 | 6 |
| Mrs. Magill ... | 1 | 1 | 0 | Nurse Twamley ... | 1 | 0 | 0 |
| Nurse Talbot ... | 0 | 10 | 0 | Nurse Fairley ... | 0 | 4 | 0 |
| Nurse Parrish ... | 0 | 10 | 6 | Nurse Crosland ... | 0 | 2 | 6 |
| Nurse Tritton ... | 0 | 5 | 0 | Nurse Stephenson.. | 0 | 5 | 0 |
| Nurse Rodgers ... | 0 | 2 | 6 | Nurse Price ... | 0 | 10 | 6 |
| Nurse Martindale... | 0 | 2 | 6 | Nurse McCord ... | 0 | 5 | 0 |
| Nurse Ryder ... | 0 | 5 | 0 | Nurse Smith ... | 0 | 2 | 6 |
| Nurse Bryson ... | 0 | 10 | 0 | | £8 | 11 | 6 |

The total received is £28 7s., a sum very much less than was anticipated.

### "A CHARITY WHICH IS NOT A CHARITY."

UNDER this head a correspondent writes: "In a cathedral city in the North of England is a district nursing association supported by the public subscriptions of the charitable, and managed by ladies. For eleven months in the year the nurses of this association bestow their kind attention on the sick amongst the working classes, but when they take their holidays these nurses all go away together and leave no substitutes to carry on their work. Reasoning with them is of no avail. The answer which has been given to the question why this is done is, 'Oh! they will learn to appreciate us more during our absence!' Here is a charity which is carried on for eleven months in the year, but during one month the sick and suffering are made to feel, and perhaps acutely, the blank which is left when the nurses turn their backs on them. Can your readers tell us of a like instance where a charity is not a charity?" It should be arranged for the nurses of the association in question to take their holidays at different times.

### SHORT ITEMS.

THE Secretary of the Kilmarnock Nursing Association has received a legacy of £25 from the late Mrs. Paxton, Blacket Place, Edinburgh, and one of £9 from the late Miss Morton, of Morton Place.—The annual report of the Jarrow Nursing Association shows that 2,498 visits were paid last year, some of the cases being of a very painful nature. Unlike some organisations, the association is so liberally supported by the townspeople, that the balance in hand at the end of the year is £46.—Miss Amy L. Jones has been accepted for the Army Nursing Service Reserve, and has been ordered to proceed for duty to Edinburgh Castle. From 1893 to 1896 she was in training at the Kidderminster Infirmary and Children's Hospital, and has since been on the private staff of the Huddersfield Nurses' Home.

### Lectures on Medicine to Nurses.

By H. A. Latimer, M.D. (Dunelm), M.R.C.S. Eng., L.S.A.Lond.; Consulting Surgeon, Swansea Hospital; Past President of the South Wales and Monmouthshire Branch of Brit. Med. Assoc.; Past President of the Swansea Medical Society; Hon. Life Member of the St. John Ambulance Association, &c.

### SOURCES OF BODY-HEAT (*continued*)—ITS REGULATION. — FEVER HEAT. — USE OF THE THERMOMETER.

I was interrupted in my last lecture, when I was saying that body-heat is the result of combustion of foods and tissues by oxygen. Another source of it is the friction of one part against another which is constantly going on within us as the result of movements of muscles. I call you to note that the same things which will produce heat outside the body, in inanimate nature, produce it in our complex system. If you briskly rub two sticks against each other, or create friction between two metals, heat is evolved; if you set a light to combustible materials, and thereby cause a chemical combination of oxygen and this matter, you create warmth; and the friction of one part against another in the body, and the union of the gas with the combustible materials of which we are formed, is equally the source of heat. Once formed, the warmth is distributed over the body by means of the blood-stream, which permeates every part of it. But just as it is necessary that there should be a producing cause, so there must be some mechanism by which an excess of it may be prevented, or by which it may be got rid of when necessary. I think there can be but little doubt that our central nervous system possesses a heat-controlling centre, the function of which is to automatically arrange for the maintenance of an equilibrium of heat when we are in health. In some diseases this heat-centre is presumably deranged, and the essential feature, of fever rise of body-heat, results. The actual mechanism whereby heat is lost is known well enough, and the parts which are concerned in this work are well recognised. The skin and lungs are the chief agents in the matter, and it is by controlling the fine blood-vessels at those parts that the work is effected. If these blood-vessels become contracted less blood is present in them at a cooling surface, and so heat is preserved in the interior of the body, for the volume of blood must be present somewhere, and if it is sparingly in the vessels of one part it is abundant in the vessels elsewhere. If these skin and lung capillaries are dilated, more blood is present in those cooled parts, and there is consequent loss of heat. Also the sweat, and the moisture exhaled from the lungs during the acts of breathing, are great agents in effecting withdrawal of heat—probably the principal ones. Everyone has experienced the coolness which follows the act of breaking out into a perspiration when in a state of dry heat from exertion.

When the blood is deranged by the changes wrought in it by the poisonous organisms of the infectious diseases, by the micro-organisms which set up the septic surgical fevers, or by inflammations of organs within the body, a derangement of the body-heat follows, and fever makes its appearance upon the scene. The predominant symptom of this affection is a rise of temperature. This increased temperature of the body has to be carefully observed, for each disease has its peculiarities in respect to the way in which fever-heat first shows itself, the range which it maintains during the illness, and the mode of its decline. In some affections—as, for example, in acute pneumonia—the patient will pass suddenly from a condition of seemingly good health into one of serious illness, and the temperature will suddenly mount to a high degree; in others—as, for example, in enteric fever—the rise of body-heat will be in a gradual manner. Then, again, there are peculiarities to be noted during the continuance and at the termination of the fever, peculiarities which I will draw your attention to when lecturing on the separate diseases themselves at a future date. What I desire to emphasise now is that very important information is derived from the use of the clinical thermometer, not only as to the progress of an illness, but as to its very nature. So it behoves you to be very careful in the way in which you make your observations of it, with a view to getting correct results.

I have been practising medicine long enough to have seen the general adoption of the clinical thermometer by medical practitioners. What was, in my earlier days, an instrument of occasional use only, has now become one of constant application in the hands of men who aim at being in any way thorough in their work. Men are becoming more and more reliant upon instruments in helping them to discover the nature of disease, as the value of these instruments become increasingly apparent. In the old days, before the invention of the stethoscope, or the introduction of the clinical thermometer, physicians formed a diagnosis by careful observations of symptoms, by examinations of expectorated matters, of the tongue, and of the pulse. Such careful examinations are as necessary now as they ever were, if perfect opinions are to be formed on an illness, especially as to the prognosis, or decision as to how the illness will terminate. But it cannot be doubted that we have made important advances in knowledge as the result of the introduction of instruments of diagnosis. As nurses you will be much relied upon in the taking of temperatures, and in the careful recording of them on charts. You should know where to place the thermometer, and should be careful to see that it has really done its work. Formerly it was customary to place it in the arm-pit, but the results of doing so were so frequently fallacious that no one who desires to have a truthful observation now allows it to be placed there. It used to be held that if the patient were fairly stout, so that the instrument could be really surrounded and gripped by the flesh of that part, the observation would be a correct one; but it was found that such was not the case in practice. I have not put a thermometer in the axilla of any person for some years past, having desisted from doing so by finding the practice valueless on two or three notable occasions when, having removed it from that situation with a low record, I found a fairly high one marked after having placed it in the mouth. This is the place in which it should be placed on all ordinary occasions in adults, and when it is put there you should be careful to see that its bulb lies comfortably ensconced under the tongue. Do not let the patient put it there himself if you want to have a reliable record. If you do so you will find that he is apt to be careless in the matter, or even, by design, careful to try to mislead you. It is funny to observe how some people will try to mislead their doctors. They appear to do this sometimes from "sheer cussedness," at other times from a desire to obtain some relaxation of diet or of regimen. A patient who feels pretty well, but is kept in bed on account of fever, and who desires to get up and be about a ward, notes that his detention is the result of thermometric records, and he sets himself to work to "cook" these records by shifting the instrument from under the tongue into the middle of the mouth, when it is cooled. Another who is convalescing from enteric fever notes that he is kept upon a restricted diet because he still has some feverishness; he craves for a change of food, and is told that he must not have it until his temperature has resumed its normal range, and so, if he can get the opportunity, he takes care that this shall be so. These are not hypothetical cases; they are ones which have occurred. Before using the instrument be careful to shake its index well down below the normal point; a subnormal temperature is as important a thing to notice as is an elevated one.

### Private Nursing in the "Golden West."

By a Sister on the Spot.

Having lately had several inquiries from nurses as to the advisability of setting sail for the "Golden West," for the purpose of "hanging out their shingle," it may interest any others contemplating a like move to have an idea of what to expect, and what is expected of them. Of course, the actual nursing is the same all the world over, more or less, and the difference consists largely in the surroundings. Some time back, in *Punch*, there was an account of a steward going to a horse-dealer to purchase a horse. He said, "That horse must do for his lordship to hunt, to draw my lady's brougham, be gentle for the young ladies to learn riding, and for the young gents to hack, and to be put to plough in the spring," whereupon the horse-dealer, with mild sarcasm, asked, "An' do 'ee want that blessed 'oss ter wait at table?" I expect some nurses will feel inclined to wither me the same way, when I begin as gently as I dare, to enumerate what is required of a nurse out here when occasion arises.

### Some Necessary Qualifications.

A nurse must be a "Jack of all trades" and "Master" too, on account of the scarcity of "help," as service is called. I have had many cases where, in addition to caring for my patient, I have had to milk a cow, feed chickens and pigs, care for a horse, and, if necessary, to harness and drive or ride them. Not only to be nurse, dairymaid, stockman, and general servant, but sometimes to be lawyer, parson, and even undertaker and sexton when those who generally fill those offices were too far off to get to. In two cases I have had to do my own "undertaking"—making the coffins, burying the dead (both children), baptising them, and reading a service over them.

### A Personal Experience.

I am just home from a case of a kind which may point a moral and adorn a tale. A short time back, being run down, I went to see one of my doctors, a jolly little Irishman with one eye.

"I've just the thing for you," he said. "You remember that case of Mrs. K——. She does not pick up her strength as she ought, and I want to try what mountain air will do for her. Her father and mother have a homestead in the 'Big Oak Flat' range, and as it is a spot remote from any habitation, she would, I know, like you to go with her as a friend. She does not need nursing; only a little care. You will have nothing to do but keep her cheerful and help her with some simple treatments."

So he arranged it. Owing to it being a place abounding in poisonous snakes, lizards, and all kinds of zoological horrors, I invested in leather gaiters, short skirt, &c., and, topped with a Mexican sombrero and hands in leather gloves, I started with my party. A lively little broncho, with a cowboy saddle on it, was led forward for me to mount at the foothills. As I was not used to mountain driving enough to conduct a team over such a road, the rider and I exchanged seats, so that my patient might have as little jolting as possible. Though more accustomed to ride sideways, I made the best of my situation and was soon astride. When all the cushions and bolsters had been adjusted round my patient we started. There was not one cushion too many. As it was, the jolts and jars when going over granite boulders or fallen trees were very trying. In the mountains anything that comes handiest and won't wear out is used for repairs, so that the roads are awful. But somehow the vehicle righted itself each time when things looked at their worst. As for my horse, it was, in appearance, like the one we see in pictures that Don Quixote bestrode. But it climbed like a goat, and, finding it knew far more about the road than I did, I did not dispute its wishes in any way, but just held on when necessary. I was glad when I was able to deposit my patient in a nice clean bed, and when she had rested a few days she seemed to pick up well.

### An Awkward Situation.

Then, when the mountaineers had started to obtain supplies, symptoms quite foreign to her previous trouble set in. Happily I had brought my hypodermic outfit, also a few emergency necessaries, as there was no possibility of getting to a doctor. By the third day fæcal vomiting had arisen, and now I had no longer any doubt that the obstruction was very serious. At last the men came back, and I got them to catch two horses, and went with one of them some fifteen miles of the roughest, yet most beautiful country I was ever in. Our mountain was "Cherokee," and we had to wind in and out and round it till I got hopelessly mixed as to the points of the compass. Arrived at the telephone I found that the doctor could not come, but would send the necessary remedies, and some only to give in case of the worst. Next morning, the remedies failing, I sent a message to the telephone to say that I felt sure there was no hope except in an operation. Still he could not come, but a regiment was stationed at "Three Rivers," and the army doctor was sent out.

### The Army Doctor.

When I heard this, and pending his arrival, I had mixed feelings, as my patient was a very weak, nervous little creature, and the army doctors here have a reputation for being rough. However, my mind was soon set at ease on his arrival. He was an excellent representative of his profession, and, when we got chatting over our dinner, he told me he had dreaded to find a "know-all" old woman up here—though my Irish doctor had said I was a "rattling fine nurse"—and was relieved to find me so amenable to orders. "That, all of you Britishers are, somehow," he said, and went on to tell of the hospitals he had been connected with—manned principally by British or Canadian nurses, who were invariably a success, &c. In the absence of another doctor I was invited to "consult," and as there was no chance of the patient's life being saved without an operation, and it was not possible to have one where she was, laparotomy under the circumstances being out of the question, we decided that the best hope for her was to make an ambulance and transport her to town, forty-five miles away. The chances looked slim indeed, for, as Surgeon Stoury said, "I hardly think she will live to reach town, but we must do something." So, turning carpenters, we soon had a spring mattress, wired, roped, and nailed on to the waggon, and a very cleverly-contrived ambulance it was.

### Charging a Syringe by Match-light.

Depositing our patient on it, on a couple of feather beds, our cavalcade started. For the first two hours the army surgeon and I rode at the back; but as darkness came on he had to leave to rejoin the regiment, and I had seven more hours, made worse by the darkness, and to administer whatever heart stimulants or opiates were needed, charging my syringe by the light of successive matches, and a tin cup. I had eaten nothing since twelve noon, and we arrived at a quarter to two a.m., so I was painfully hungry and thirsty. The doctors had everything ready, and the operation was performed, and has proved a success. Of course, the cases are mostly as prosaic as in a little country town, but one has to be prepared for anything and to shirk nothing. The climate is very hot in summer; as I write in the shade in my patient's room I can see it—110 deg. In winter it is frosty at times, and foggy and damp. Spring and fall are glorious. The orange and lemon groves, peach, apricot, and all kinds of fruit are grand, and the mountains more so.

### An Opening for Nurses.

There is room for another nurse, or even two or more if they will take obstetric cases, in this town of Visalia, Tulare county, and, I believe, in many other towns in California, for I have more than I can do here. I was for a long time the only trained nurse working in Tulare, which is a tremendous strip of country if it is examined on the map.

### The Question of Finance.

The pay is from 15 dols. up per week, which is equal to about £3 3s. of our money. In the big cities it is from £4 4s. Living here is very cheap, and where two or three nurses co-operate they can do well, and save a considerable sum yearly. The doctors are very pleasant to nurse for, and after their first case have the most unbounded confidence in the nurse, which makes one try all the more to deserve it. The patients are, as a rule, more nervous and fidgetty and exacting than those in England, but otherwise the household generally bows to the nurse's rule, and is grateful and willing to help. A trained nurse takes as good a position as any one in town, if she is a lady in manners and education. This valley is fairly salubrious, but malaria and typhoid fever are by no means uncommon. For any not able to stand severe English winters it is excellent.

### Nursing the Sick and Wounded in China.

By a Hong Kong Correspondent.

Hong Kong, in spite of its importance as a naval and military station, is unable to supply nursing sisters "for the front," simply because these departments do not possess them. More than that, there is no accommodation provided for military officers, who are either attended in their quarters or are sent to the Government Civil Hospital to be nursed. From the latter source, since the need was urgent, two sisters —Miss Barr and Miss Batchelor, of the London Hospital—were handed temporarily over to the naval authorities for their base hospital at Wei-hai-wei, where Miss Gladwell, of King's College Hospital, helped them for a few weeks. Since the latter's return to Shanghai, three Corean nurses and Miss Vaughan, from India, have augmented the staff, and five others, viz., Misses Lingard, Hill, Waterhouse, Cusins, and Hislop, are *en route* from the same source, the first two being due in the "Carthage," recently fitted in Bombay as a hospital ship. There have been many volunteers for nursing work from missionary and other sources; later, when another base is established, as proposed, at Yokohama, selection may be made, but this is uncertain.

### The Native Nurses.

As to the amahs—native nurses—in Hong Kong, they enliven the playtime of their charges by telling them "Soon there will be no white faces left; all must makee die Others demand to go to their homes in Canton before fighting begins, and are only appeased by an increased wage—the panacea for most Chinese ills.

### The Wounded at Wei-hai-wei.

It is a pity that Wei-hai-wei cannot accommodate more wounded, for the first batch—seventy odd—certainly does credit to its health-giving air. The men all seem cheerful, and tell of hair-breadth escapes from shrapnel, shell, and bullet. One man when lying down firing was shot in the shoulder, and the bullet found exit at his thigh. Another pierced the liver from the front and travelled out at the patient's back, while a less fortunate comrade lost the sight of both eyes from a bullet lodging in his forehead.

### Japanese Help.

The Japs have done much in the way of helping with the sick and wounded, and we are indebted to this sturdy, clever little race for more than one marked kindness. Now we ought to be able to dispense with their help, for field hospitals and hospital ships promise ample accommodation, and are supplied with all that is fittest and best, offers of luxuries and extra comforts having already been tendered by those at home and abroad. The fund for "Our Soldiers and Sailors," started some time ago, swells daily, and is a further proof of the never-failing Colonial generosity.

### A Royal Present.

The Emperor of Corea sent down for the allied forces numberless boxes of cigarettes together with thousands of bags of rice and flour, a most welcome addition to the present fare of tinned meats obtained at abnormal prices. Foraging parties go out daily in search of supplies, and welcome is he who brings in the patriarchal chicken—despised as only those who have lived in China can despise it—which now proves a toothsome morsel.

### The Peril at Tientsin.

A letter from Tientsin to-day tells of the noble disregard of personal danger upon the part of those who go on to the field to succour the wounded, and one realises more what this means when one is told "to stand up would be fatal, in such a hail of bullets; the wounded are dressed by the surgeon, who lies side by side with his patient (imagine the difficulty of this procedure), and when this task is completed, the good Samaritan crawls on all fours to the next in need of his aid, and so on and on through the blazing heat of a tropical day, until it is followed by the welcome dark, under cover of which the wounded are carried into hospital."

### Nurses' Reading Society.

The first year of the society having ended in July, Miss Moberly is now able to announce the names of the winners of prizes for the year's examination questions. The answers to questions set in June have just been corrected. The following are the names of the prize winners: First prize, £1, Miss Maude Smith, 380 marks; second, 15s., Miss Jessie Till, 375; third, 10s., Miss Osyth Wilson, 365; fourth, 10s., Miss Boraston, 360; fifth, 5s., Miss H. Deudy, 340. Miss Moberly wishes again to remind members who joined in July, 1899, that their subscriptions are due. She states that a very large number of those who joined at that date have neither sent their subscriptions nor intimated any wish to leave the society. The books set for next quarter are "A Manual of Obstetric Nursing," vol. I., by M. Humfrey; "Nursing of Diseases of Nose, Throat, and Ear," P. Macleod Yearsley. Miss Moberly proposes, at the end of six months, when these books have been read by all the nurses, to set vol. II. of the "Manual of Obstetric Nursing." She thinks it is better not to set both volumes in the same quarter, as nurses may not care to have so long a "run" of the same subject.

### Nursing at No. 6 General Hospital, Johannesburg.

A member of the Army Nursing Service Reserve writes us: After a long and very curious journey from Naauwpoort to Johannesburg, we are very comfortably established here in our new quarters. We travelled in No. 3 Hospital train, not as nurses but as passengers, and a very curious experience it was. The country after leaving Kronstadt was exceedingly interesting. Nearly the whole way along the line has been blown up at some time or another by the Boers, and I do not think there can be a single original bridge left; it is quite sad to see them all broken down and lying by the side of the line which is now used. For miles and miles on either side the line there is nothing but flat grassland quite bare, either burnt by the Boers or trampled down by the marching of troops. It makes one's heart ache to see numbers of dead horses and mules, and occasionally oxen, lying along the route. Every place is fortified, and some of the earthworks are very wonderful and interesting.

### Not Allowed to Travel at Night.

One night we spent at a funny little wayside station. We were not allowed to travel at night, because it was not considered safe, and only at the rate of five miles an hour in the daytime. At this little station there were sentries all along the line to guard us, and all lights were ordered to be put out at nine p m. De Wet was supposed to be within fifteen miles of us, and I quite hoped something exciting might occur. However, nothing did; only several times in the night I woke up wondering where I could be, hearing the sentries challenging and the guard very suddenly turned out. A few nights after this the trains were fired upon and the lines cut.

### Arrival at Johannesburg.

After crossing the Vaal River the scenery changes and becomes much prettier. The earth looks richer, and in parts is deep red sand. Firs and eucalyptus (or blue gum trees, as they are called here) are to be seen now and again, and hills rather like our Sussex Downs. We halted for half-an-hour at Elandsfontein and then went straight on to our destination at Johannesburg. We received a very hearty welcome, and felt very happy to have come to an end of our journey. On Sunday I went to evensong at St. Mary's Church—such a nice service, everything most reverently done and very good music; it was a great treat. The church was filled with officers and soldiers. "God Save the Queen," with the band and organ, was grand; but, much as I love it, it is the one thing that makes us all feel a little bit home-sick. I have never seen anything like the mimosa here. There are huge trees of it, and they look wonderfully graceful and beautiful. There is no fruit to be had now but oranges, and they cost 6d. each. This is such a thirsty country that we should prefer being able to get them for something less than that. The warmer weather is commencing, but this morning at nine a.m., on my way to duty, I saw large icicles in the garden in spite of the sun.

### The Death Rate at Naauwpoort.

I feel very sorry so much has been written about the care of the sick and wounded. It is impossible to compare active service with the work done at home in civil hospitals, because they are two such totally different things; but, taking everything into consideration, I am sure, notwithstanding all that has been said, that the sick have been well cared for and looked after. I judge by what I have myself seen. At Naauwpoort our death rate worked out at 13 per cent., including all the bad enteric cases we had at one time, and I am sure that is not high.

### Under Canvas.

Our hospital is all under canvas, with the exception of the officers' quarters, and very glad we are, now the warm weather is commencing, not to be in buildings. We have become so accustomed to an outdoor life that we much prefer it to an indoor one, and I am sure out here it suits the patients better too. The hospital is situated in the grounds of the Wanderers' Club, and we could not have a better site. It is enclosed by wooden palings, and inside these are eucalyptus trees, planted all round. It is a pretty camp. There is one large hall which is being prepared as a recreation room for the convalescents, and it will be just delightful for them. We are having a very good theatre built, with a Röntgen room attached to it. It will have a concrete floor, electric light, and I know not what else, but everything that is needed to make it as nice as possible.

### The Surgical Division.

I am once more, to my great delight, in the surgical division. This is on rather a higher part of the ground than the medical division. The enterics are being nursed in another part of the grounds. The hospital is all well planned and arranged, and will be extremely nice when it is quite in order. At present we have hardly had time to get things straight. In our division we have under our care only three marquees, with seven patients in each one. I hardly know myself with so few patients to look after, though I never mind having plenty of work.

### Amusements.

There was a cricket match on the bicycle track yesterday, which is also in the hospital grounds, Military *v.* Civilians. The former won by three wickets and 120 runs. One of the regimental bands played, and we had tea in our officers' mess. The cricket pitch was Indian matting. One day we drove out to the place where Lord Roberts and his staff stayed. We saw "Otto," the little boy he taught to spell, and whose picture was in the illustrated papers. He was very shy, but was pleased with some chocolate I took specially for him. We had an excellent tea on the verandah, and heard the praises of "Bobs" sung in every quarter. The soldiers simply love him, and I do not wonder after all one hears of him. Lady Roberts is going round our hospital very shortly, and I hope Lord Roberts will come too, I want so much to see him. The drive to the inn was very pretty, though only two miles out of the town; hills in the distance, plenty of trees, mines in sight, and mimosa trees smelling as sweet as they looked.

### A Dead City.

Johannesburg itself is like a dead city. Nearly all the shops are closed, those that are open have next to nothing in them, and what they have is an exorbitant price, as the poor shopkeepers have no hope of replenishing their stores before Christmas. Butter is 7s. 6d. a pound, so we do without it; fresh milk is almost unknown, and condensed milk is 1s. 6d. a tin, and so on, but our sister in charge is a splendid housekeeper, and we manage very well.

### Where the Sisters Live.

We sisters are living in three furnished houses, all commandeered, of course. Ours is very comfortable, and, indeed, after living in bare rooms so long the magnificence of my surroundings so oppressed me the first night that I could not sleep! There is a large verandah leading out of the dining-room, where we hope occasionally to have tea, and another verandah leads out of one of our bedrooms, and we spend a good deal of our off-duty time on it in our deck chairs. The garden is not very large, but it is full of roses and violets. We breakfast at eight a.m., go on duty at nine a.m., lunch at one p.m., are off duty in the afternoon, go on duty again at five p.m. or six p.m., and dine at half-past seven, so our day is not a hard one. I am trying to get a bicycle, but the Boers have been beforehand and commandeered all the ladies' bicycles. You see, having already told you how many privileges and luxuries we have here which we have not had hitherto, I am ending up my letter by wishing for yet something else. But it would be so very pleasant to ride about and explore the country.

### Nursing in the Open Air.

### A VISIT TO ROSTREVOR SANATORIUM.

By a Correspondent.

One of the principal trials of a nurse's life is the small amount of fresh air that her duties permit her to enjoy. In many hospitals there are only two, or perhaps three, "off times" during the week for the majority of the nursing staff; so that four or five days out of the seven are spent entirely indoors. This is not quite so trying to the health as the general public imagine, because the life itself is so regular and hygienic as greatly to minimise the ill-effects of insufficient open-air exercise. Still, all nurses know the craving for open breezes and free space that comes at times to the patient workers in the wards; and to these some account of "open-air nursing," as it is carried on in many of the new consumptive sanatoria, will be interesting, if a little tantalising, during the present bright summer weather. As an example, the Rostrevor Sanatorium, which I recently took the opportunity of visiting, is specially attractive, owing to the beauty of its situation and the fact that Rostrevor has long been known as a place particularly suitable to phthisical patients. The little town of Rostrevor lies on the shores of Carlingford Lough, County Down, not far from the well-known steamer station of Greenore. The Sanatorium, which is situated in the heart of the beautiful Mourne Mountains, some 300 or 400 ft. above the town, is conducted on a combination of the well-known Nordrach and Falkenstein systems, but differs from most of the Continental sanatoria in the matter of nursing. The physician who owns and manages the Rostrevor institution is of opinion that the constant care of trained nurses is essential to the welfare of all phthisis patients. A brief sketch of the duties expected from the nursing staff of such an establishment will convince most people of the truth of this pronouncement.

### Beginning the Day.

At half-past seven the day's work begins. Milk, hot or cold, is carried round to all the patients before they rise. Some sleep in the main building, which is connected with the physician's house by an open passage; others, in the pretty little "sleeping bungalows" dotted about the grounds under the shelter of tall, fine trees, or among huge rhododendron and laburnum clumps, looking far away across the peaky purple hills to the silver streaks of sea that shine here and there through long, green boughs. All the bedrooms have detachable window-frames, which are (practically) always kept out, so as to admit unlimited air. The patients are well wrapped up, and never complain of cold. After the milk is disposed of, every patient is thoroughly rubbed with methylated spirit, to harden the skin and protect against chills. Some are also massaged, in various ways. Temperatures are taken three times a day.

### An Important Task.

Before the half-past eight breakfast the milk and butter allowances for the day are measured out by the head nurse. This is an important task, as every patient is on a special allowance of butter and milk (no cod liver oil being used), and must dispose of a certain amount during the day. The butter is placed in pretty china dishes, each bearing a name label, and appears on the table at every meal. The milk is given out at stated hours during the day; some patients, who are unable to take much solid food, having milk every hour. The diet is very liberal and tempting, but patients, although they are generally coaxed into eating a great deal more than they could manage at home, are not forced to swallow solid food in quantities from which their stomachs actually revolt, as is done in some of the foreign sanatoria. Many patients take their meals lying on their couches; to these the nurses go round, also attending to those who are in bed, but they do not lay tables or wash up. After breakfast the physician sees the patients and gives the nurses their orders. There are very few medicines to give out, as a rule, diet being the backbone of the treatment. Orders are also given as to the disposition of the couches for the day. "Strong winds are poison," as Brehmer says, and the patients lie out in the open so many hours every day that situations have to be carefully selected, according to the weather. During the winter time, or in very windy weather, their couches are generally set in the gardens, where high beech hedges and groves of trees shut out the gusts that sweep rudely over the open lawns. On breezy days the revolving shelters, or "Liegenhalle," come into requisition, and during rain the open verandah is used. Patients whose temperature is too high to permit of their getting up can have their beds run out into the verandah, or else they can lie at their own open windows, from which the detachable frames have been removed. While the patients are taking their morning exercise (of the length prescribed for each), the nurses collect the sputum cups used during the night, also the jars which hold the patients' handkerchiefs while they are in bed (during the daytime washable and detachable pockets are used). The sputum is measured, carefully examined for traces of blood, &c., and left for twelve hours in carbolic (1 in 20) before throwing down the drains. The nurses are at present being taught to stain for tubercle bacilli.

### Dusting and Disinfecting.

The dusting of the main building is a serious business. Although the sanatorium is three miles from a village, near a quiet road where no dust arises, all the floors, passages, and furniture are wiped over every morning with dusters wrung out in 1-20 carbolic, or some other powerful disinfectant. The walls (of varnished pine) and ceilings are also frequently wiped over. No sweeping is allowed. The furniture of the patients' rooms is specially made, without mouldings or turnings where dust might lie, and the heavier pieces stand high on easy-running castors. Untrained assistants, working under the nurses' eyes, are employed to help in this long job. The day is occupied in further serving of meals, giving out milk, taking the patients for quiet walks at a slow pace, disinfecting bed-linen, handkerchiefs, and pockets for the wash, &c. The nurses are, of course, responsible for the carrying out of the doctor's orders; and, as phthisical patients are often freakish, fretful, and obstinate, this is no light task, in the case of a system comprehending so many details.

### No Night Nursing.

Each nurse has two hours off duty during the afternoon or evening. One sleeps in the patients' building, her room being connected by electric bells with all the other rooms, and also with the doctor's. There is practically no night duty, as a "special" is always wired for if any patient is in a sufficiently serious condition to call for night nursing. In the evening, six o'clock tea and a quarter to nine o'clock supper have to be attended to. The patients are in bed at nine, and most are rubbed a second time or massaged at this hour. The sputum cups and flasks which have been carried by the patients during the day are now collected and changed, as in the morning. Once a week the physician in charge weighs the patients, and the head nurse keeps note of the results. When a patient leaves it the room is thoroughly fumigated with crude carbolic and the bed carbolised.

### Interesting and Pleasant Work.

Although not exciting, nursing in an open air sanatorium is interesting, healthy, and by no means over-fatiguing. It is work best done by educated gentlewomen, thoroughly hospitaltrained, demanding, as it does, character, decision, patience, conscientiousness, and good manners on the part of the nurse. Young girls are often taken as probationers, without premium. The salaries given to trained nurses are generally good. In the Rostrevor Sanatorium the nurses take their meals at the doctor's table. Their bedrooms are separate, and are exceptionally pretty and pleasant. Like every other room (including the bungalows) they have electric light. As the nurses themselves partake of the benefits of the open-air system and the liberal feeding, they find the life an extremely healthy one, and do not suffer at all from the strain and fatigue often inseparable from the routine of a general hospital. It is a life that one can recommend to any nurse for whom the "burden and heat of the day" in a great town are proving too much; and, owing to the recent multiplication of consumptive sanatoria, many posts of this kind are now obtainable. Untrained or partially trained nurses are seldom accepted as "staffs" in these places, as previous general training is really necessary. Would-be probationers will usually find some little hospital experience an advantage in applying, but there are many sanatoria which take entirely untrained girls of good education, and a year or so of sanatorium life is no bad introduction to the career of an ordinary hospital nurse.

### A Picnic for Sick Children.

By a Correspondent.

Some of the readers of The Hospital "Nursing Mirror" may have heard of the Cottage Hospital for the Surgical Treatment and Nursing of Children, suffering chiefly from hip and spine diseases, at Coldash, near Newbury. It stands on a fine piece of tableland, five hundred feet above the sea, interspersed with pine woods whose delicious scent, together with the purity and the dryness of the air, is especially good for the little people who come in a large proportion from the East End of London sent down by the Children's Aid Society. The other day an unusual pleasure was given to the inmates in the shape of a picnic to celebrate the birthday of the founder and superintendent of the hospital, Miss Bowditch. It was a warm afternoon, and most of the little folk were out of doors, some under the tent, others in their little wheel carriages holding up sunshades of varied hue and age, and others again promoted to crutches, hopping about with much cheerfulness. All, of course, were talking about the picnic. "Will it keep fine?" and heads were turned up to the blue sky, where there were certainly a few clouds. "Will it be too windy?" asked the blue-clad nurses, who displayed even more than their wonted activity. But as the day advanced the weather was pronounced warm and safe enough for every little person to be out and away. And so, at three o'clock, all patients, nurses, and attendants took the road, and the building was locked up

A quaint procession it was on the clean, sandy road leading to the woods. Well ahead were the boys and the girls who, with the aid of crutches—a select minority were without—covered the ground at a great rate. Behind came the donkey and pony carts—the latter lent by a kind neighbour—with their freights of little ones. Then followed sedate girls on foot, holding the arms of favourite nurses, and bringing up the rear were nurses in charge of the helpless ones stretched on their carriages, but with alert, expectant faces. All but one, a tiny, pale-faced girl of ten, who looked only six, with a sad, pathetic face. It is that of a child rescued by the Society for the Protection of Young Children, and sent to Coldash from University College Hospital.

Presently the donkey and pony carts, and all the little company passed through a gate into the lovely wood. The air was sweet—pungent and yet balmy—and happiness reigned supreme as the party broke up into various little contingents. Cricket-loving boys found a cleared space, and with wonderful deftness played a modified form of the national game. Carriages of smaller size were wheeled by gentle nurses up and down the woodland paths, fragrant with the odour of the pine needles. The pale-faced child had her lap filled with acorns, gathered from a glorious oak. Little Florrie, aged three, with blue eyes and swathed legs, toddled along in most enterprising fashion, gathering a posy of heather for "Miss Bowdie." Lying on a bed of heather, and half hidden by tall bracken, were two fair-haired little girls weaving crowns of heather.

Meanwhile, busy hands had been spreading the white cloth on the ground and covering it with cakes and buns and fruit; conspicuous in the middle was a large sugared cake, given by a kind lady friend, who later on dispensed it bountifully. Scouts were sent out to gather the party together, and in a very short time, considering the weak backs and legs, a circle was formed round the white tablecloth and Miss R.'s fine voice led off with "Be present at our table, Lord." Attendant maids brought hot water from the nearest house and cups and mugs were handed round. Cakes, &c., were eaten galore, and the summit of happiness was reached when the "Buszard" was cut into. The treasurer of the hospital, who had given up some of his time that afternoon, passed it round with little jokes which produced much innocent laughter. "Round the Mulberry Bush" was enjoyed by many. The boys rushed about getting hot and excited; the little lying-down girls played a sedate game of ball with frequent "misses"; and finally all stragglers were brought in, another circle was formed, and Miss R. sang "I Once had a Sweet Little Doll, Dears," to which even the boys, tired and subdued no doubt, listened respectfully. It was a pretty sight. The children, the blue and white nurses, the feathery larches, the trembling, silvery birches, all touched here and there by the low rays of the fast sinking sun. Then came the packing and tucking up, and next day not one of the patients was a jot the worse for the September picnic.

### Appointments.

Llandudno Infectious Diseases Hospital.—Miss Winifred L. Williams has been appointed Matron. She was trained for three years at the Carnarvonshire and Anglesey Infirmary, and for two years at the St. Marylebone Infirmary. She has also had twelve months' fever training, and for eight months has discharged matron's duties at the Carnarvon Fever Hospital. For twelve months subsequently she was at the Hanover Private Hospital, Hanover Square, W.; for three months at St. Mary's Lying-in Hospital; and for twelve months she held the position of matron and lecturer at St. James's Nursing Institute and Training School, Walthamstow. She has the L.O.S. certificate.

Bradstock Lockett Home for Sick and Cripple Children, Southport.—Miss A. K. Gough has been appointed Lady Superintendent. Miss Gough was trained at the Royal Infirmary, Bristol, and has been matron of the Hampton Court Cottage Hospital, matron of the Freshford Cottage Hospital, and superintendent of district nursing, and, for the past nine months, matron of the Dawlish Infirmary. Miss Gough holds the L.O.S. certificate.

Cork Street Fever Hospital, Dublin.—Miss M. Isabella McRae has been appointed Assistant Lady Superintendent. She was trained for three years at the Royal Albert Edward Infirmary, Wigan, where she has since been sister and night superintendent. For the last eighteen months she has been night superintendent at the Grimsby and District Hospital.

North Staffordshire Infirmary.—Miss C. A. Padbury has been appointed Night Superintendent. She was trained at Leicester Royal Infirmary, and has been charge nurse at the Fountain Fever Hospital, Tooting, London.

### The Nurses' Bookshelf.

[We invite Correspondence, Criticism, Enquiries, and Notes on Books likely to interest Women and Nurses. Address, Editor, THE HOSPITAL (Nurses' Book World), 28 & 29, Southampton Street, Strand, London, W.C.]

THE HUMAN FLOWER. By ELLIS ETHELMER, author of "Woman Free," &c. Fourth Edition. Revised and Enlarged. (1900. Price 1s. 1d., from Mrs. Wolstonholme Elmy, Buxton House, Congleton.)

THE purpose of this book is, as explained by the author in her preface, "to give in plain and inoffensive terms a short and simple account of the circumstances connected with the birth of human beings into the world" She is to be congratulated on the success with which she has performed a difficult and delicate task. Every one is convinced that both boys and girls, at the age of puberty, should know a great deal more about their bodies and the functions of each organ than they generally do; but everyone must sympathise with the reluctance of parents to enter into the discussion of the most delicate questions of physiology with their children. Sometimes the children ask questions, and are put off with an evasive answer; but as often as not an instinctive modesty keeps them from making any inquiry, at least from their parents, regarding a subject which, nevertheless, must be occupying their minds, and if they get any information at all, it is conveyed secretly, and with more than a suggestion of pruriency, by companions of their own age. Parents who are conscious of the importance of accurate knowledge of the responsibilities of manhood and womanhood being gained by their children, yet feel themselves unable to give it with due delicacy and discretion, cannot do better than put Ellis Ethelmer's pamphlet in the hands of their sons and daughters. Beginning with the phenomena of the fertilisation of a flower, the author goes on to the analogous process of the fertilisation of animals and man, showing how the one process is as natural and as legitimate as the other, and how desirable it is that the latter as well as the former should be well understood and carried out with full consciousness of the probable results. That this little book has reached a fourth edition shows how real the necessity for such a manual is, and it cannot be too widely known.

USEFUL WORK FOR GENTLE HANDS. An Address on Laundry Work as an Occupation for Gentlewomen. By J. T. BUDD, Second Edition. Enlarged and Illustrated. (London: S. W. Partridge and Co., 8 and 9, Paternoster Row, E.C. 1900. Price 6d.)

THE object of this little pamphlet is sufficiently set forth in the title-page. Mr. Budd shows how many women there are who need to earn their own living and do not know to what to turn, having had no special training of any kind. He shows also that in many laundries, as well as in homes of all kinds where laundry work is done, there is need for educated and capable women to supervise the workers and do the finer work. The attempt to turn the attention of poor gentlewomen to this work is sensible and praiseworthy. There is nothing offensive in laundry work, and the getting up of fine linen and lace is almost an art, and is a task in which any woman may take pleasure. It is perfectly adapted to women of refinement. In many laundries, also, where the actual work is well done, the book-keeping is faulty; and there is ample room for women of education to attend to the sorting of the laundried clothes, and seeing that each garment reaches its proper owner. Still further work might be found for women of a superior class if mending were done at the laundry, and we believe that many housekeepers, not to mention the helpless bachelor, would welcome a suggestion that it would be done for them there. This little book certainly opens up a new avenue for the occupation of women, and it is all the more encouraging that the author can show that a creditable record of success has attended his efforts in placing ladies in laundries. We think, however, that he does not lay sufficient stress on the necessity of proper training for the work, nor tell where such training may be had. It would be very unwise to place at the head of a laundry a woman, however gently born, however indigent, however anxious to work, who cannot instruct those under her as to the different methods of treatment required by white and coloured clothes, flannels, lace, and the like, and who cannot show by actual demonstration every process, from the soaking of the clothes to the last touch of the goffering iron.

### Minor Appointments.

HAM GREEN FEVER HOSPITAL, BRISTOL.—Miss Caroline Terry has been appointed Charge Nurse. She was trained at the Royal Southern Hospital Liverpool. Since then she has been charge nurse at the Royal National Hospital for Consumption, Ventnor, has been engaged in private nursing, and lately has been charge nurse at Park Hospital, Hither Green.

EAST POORHOUSE HOSPITAL, DUNDEE.—Miss C. M. Nicol has been appointed Charge Nurse. She was trained at Leith Hospital, and has since been staff nurse at Belvidere Hospital, Glasgow, charge nurse at Eastern Hospital, Homerton, Northern Hospital, Winchmore Hill, and Park Hospital, Hither Green. For the last year she has been doing private nursing at Bournemouth.

NORTH DEVON INFIRMARY, BARNSTAPLE.—Miss Mary Mitchell has been appointed Charge Nurse. She was trained for three years at the North Staffordshire Hospital, and for two years at the Monsal Fever Hospital. She has since been staff at the North Eastern Hospital and charge nurse at the Accident Hospital, Hebburn-on-Tyne.

COOK'S TERRACE INFIRMARY, PANCRAS ROAD.—Miss Marian Alice Bowles, Miss Louisa Annie Barker, and Miss Johanna Fitzgerald have been appointed Staff Nurses. Miss Bowles was trained at Watford Infirmary, and Miss Barker and Miss Fitzgerald at St. Pancras Infirmary.

CLAPHAM MATERNITY HOSPITAL.—Miss Frances Fry has been appointed Staff Nurse. She was trained at the West Kent General Hospital; the Hospital for Women, Soho Square (for midwifery); and Bristol General Hospital. She has been charge nurse at Bristol Infirmary.

STAPLETON WORKHOUSE HOSPITAL, BRISTOL.—Miss Julia B. Cox has been appointed Charge Nurse. She was trained at Guy's Hospital for three years, and has since been night nurse at Home Birds' West, Kingston County, Dublin.

### To Nurses.

WE invite contributions from any of our readers, and shall be glad to pay for "Notes on News from the Nursing World," or for articles describing nursing experiences, or dealing with any nursing question from an original point of view. The minimum payment for contributions is 5s., but we welcome interesting contributions of a column, or a page, in length. It may be added that notices of entertainments, presentations, and deaths are not paid for, but, of course, we are always glad to receive them. All rejected manuscripts are returned in due course, and all payments for manuscripts used are made as early as possible at the beginning of each quarter.

### HOSPITAL SERVANTS.

MATRONS of hospitals who experience difficulties in getting maids and attendants may be glad to have their attention drawn to the system which the National Domestic Association of 27, Brook Street, Hanover Square, have in force. They state that they have a number of girls from the country who are willing to accept such situations—some who have experience and others who have not—and that they undertake to carefully investigate the characters of the girls before bringing them to London. Care is taken by the association to engage only those who are healthy and strong.

### Echoes from the Outside World.

### AN OPEN LETTER TO A HOSPITAL NURSE.

Concurrently with the Dissolution of the British Parliament, it was announced on Tuesday that the first session of the Parliament of the Australian Commonwealth would be opened by the Duke of York in the name of her Majesty. Moreover, the Duchess of York is to accompany her husband on his visit to the Australian colonies, the Queen's assent being, of course, given "on the assumption that at the time fixed for the Duke of York's departure the circumstances are generally favourable as at present, and that no national interests call for his Royal Highness's presence in this country." Everyone will fervently hope that the Fates may be propitious; and, meanwhile, the news which has been flashed to the Antipodes has been received with most profound satisfaction, and, indeed, with general enthusiasm, in that distant part of the empire. Whether the happy idea originated with Lord Salisbury, or, as some say, with Lord Selborne—the Under-Secretary for the Colonies, and the Premier's son-in-law—it is the Queen herself to whom Australia is primarily indebted for the promised visit of her grandson. No surprise would have been felt if she had pleaded that, at her age, she could not spare him for so long a time; but, as usual, she has put aside her own inclinations for the benefit of her people.

---

Tidings from the seat of war in South Africa comes slowly, like water from a cistern when the supply is running short. General French's capture of Barberton, with much ammunition, stores, abundance of railway stock, and cattle in large quantities is the most important action during the last week, but all along the lines matters have gone satisfactorily. Mr. Kruger has developed ophthalmia to add to his other troubles, so it is stated, and his wife upon receiving a telegram at Pretoria to say that she was to join him at Delagoa Bay, replied that "her health was not good enough to travel." Eventually, however, she changed her mind, and they are sailing for Flushing. I cannot help thinking that had I been left in the hands of a successful enemy by my husband, as she was, my health would have suffered in the same way when he wanted me back again. There is a good deal of talk, which, happily, is no longer premature, as to what will be done at the end of the war. Amongst those who will receive the V.C. it is announced that there will be a Canadian, Sergeant Richardson, and that a certain number of officers and men, representing, as far as possible, each of the Colonial corps now serving under Lord Roberts, will be invited to visit this country as soon as it becomes possible to reduce materially the strength of the South African field force. During their stay these men will be the guests of the nation, and the Queen has intimated her desire to inspect the Colonial troops, and to present them with colours commemorative of the distinguished part which they have played in the campaign. May I be there to see!

---

Only last week I told you how Mrs. Scott, wife of the Bishop of North China, had interested herself in promoting a fund for the benefit of the widows and dependent relatives of the soldiers and sailors who perished in the struggle with the Boxers. Almost before the statement about her effort was printed came the announcement of her death. There is only too much reason to fear that the sad event, though assigned to dysentery, was partly due to the great strain endured by Mrs. Scott during the bombardment of Tientsin. Moreover, during her residence in Wei-hai-Wei she was unremitting in her attentions on the sick and wounded in the hospital, freely rendering all the assistance she could in nursing them. It is but eleven years since she was married and quitted the shelter of her father's home at Oxford to face a life of peril and self-denial in the Far East.

---

It is very evident that the lapse of years does not diminish the faith of the believers in Mrs. Maybrick's innocency. My reference to the late Lord Russell's letter has elicited an interesting communication from a Dublin barrister, who reminds me that "Sir Charles Russell's defence of the prisoner was remarkable for its extremely unimpassioned character and for its moderation and accuracy." He further says, "So true was this that on the occasion of Lord Russell's death the *Liverpool Post*, in a leading article, stated that Sir Charles Russell, finding the Judge to be utterly incompetent, resolved in the interests of justice rather to sum up the evidence than to defend the prisoner, and that he practically summed up in favour of a conviction! This was, of course, an overstatement, and had Sir Charles Russell been addressing a special, instead of a common, jury the verdict would probably have been different, in spite of the Judge's impassioned and very inaccurate speech for the prosecution. When the letter to Mrs. Maybrick was written the Judge had died, and the writer, besides being the head of the Criminal Justiciary of England, was the only Judge of the High Court who had taken part in the trial, and was fully aware of the whole case. I think you will admit that the absolute rejection of the representations of such a man—for the Home Secretary never made any concession whatever, not even the reduction of the life sentence to a term of years—is a matter which requires explanation, and the public ought to insist on having the whole correspondence laid before it, and forming its own judgment on the merits. It may possibly turn out that there is not so much a difference of opinion as a difference of methods. It seems, I think, to be now conceded that the Judge was incompetent. Ought not this fact to invalidate a trial by Judge and jury?"

---

For some time we have been threatened with the departure of the blouse. I use the word "threatened" advisedly, because we have so learnt to depend upon this useful garment that it would be a sad blow to most of us to find that it was no longer possible to wear it without incurring the stigma of being "frumpish" or "old-fashioned." It must assuredly, in the original, have been the creation of an eminently practical mind, and the busy woman, quite as much as the girl with a limited income, has had cause to bless the originator. Amongst nurses it is, I know, a great favourite, for it so economises luggage, especially for a twenty-four hours' outing. A nice coat and skirt, with a couple of blouses, enables the holiday-maker to pay an afternoon call at one house and to pass on to a little dinner at another, or to join a theatre party, still feeling herself suitably attired. Having regard to the convenience of such an arrangement, it will, I am sure, be good news to most of you that "blouses are to be as much worn as ever." This comes from a reliable, because a fashionable, source, but it needs just a little modification, or, rather, expansion. A year or two back a person buying a costume would very probably have bought a new skirt and a new blouse both perfectly distinct from each other. Now, for a walking gown a wise purchaser would have the same material for both bodice and skirt, but for indoor or evening wear the pretty silk or velvet blouse is still well to the fore. The day it departs for good many of us, I think, will go into mourning.

### "Wee Donald."

(Taken from Life.)

Dear wee Donald! He was brought in one day, a wee "Hielan' laddie," six years of age, from one of the small villages in ——shire. A hopeless case, the doctors said, as they gently examined him. It seems he had been swinging on a cart on his way from school. His heel caught in the spokes of the wheel, and he was terribly injured; but all that could be was done, and as the days went by the doctors grew more hopeful.

When I first saw "Wee Donald" he was strapped in a "coffin," or the nearest approach to one, and nothing visible but a wee brown face and hands, and such a winsome little face!—so tanned with the Highland sun and wind, but now marked with lines of pain. Very sad must have been those first nights and days to the restless little body now unable to move. But not for long. With the happy adaptability of childhood he soon learned to forget it, and what a merry little chap he was. Nothing escaped him, and he soon became the life of the ward. It was Donald's voice that heralded the dawn of a new day as he shouted to his sleepy confrères, "Hoo ye're keeping?" and as the answer came, "Hello! how's yourself?" "Oh, fine," he would say; and so through the whole day he would keep up his merry little chatter, and anon whistle like a blackbird, one of his favourite tunes being "There is a Happy Land." Then hearing the Highland Pipers as they marched past the infirmary en route for the Transvaal play "Cock o' the North" and "Will ye no come back again," he reproduced it all until at times he had perforce to be silenced, which was only effected by screening him off as one would a noisy songster.

At times the pain was bad, and on such occasions it was a wistful little face we saw, with such a desire to be petted and comforted. He was "very bad, gey sair," and the cry would come, "Nurse, sort my cord." This is unintelligible to English ears and needs translation, but "Shake my pillow" was what he meant. In a little while he would be easier and his happy little self again, the pain all forgotten.

There was a sad incident in his story. His mother came to see him, and it was noticed that she did not "mother" him as one would naturally expect. A day or two after another woman appeared and asked to see him. At first she was refused. "But I'm his mother," she said. She persisted in her statement, and at last the doctor gave permission. There was no doubt in our minds as to the truth of her statement when we saw her fondle him. It was another case of wise King Solomon's judgment. She had been separated from her husband, but still desperately loved her boy. The little laddie was good friends with his "steppy," as he called his step-mother, and I think she did her best for him.

I'm afraid we all conspired to spoil him, but it can hardly be wondered at. It seemed such a hopeless case at first. However, his native Scottish air and the breath of the highlands had given him an heritage that is lacking in his town-bred brother, and where the one would almost inevitably have succumbed, the natural vitality of this true child of Nature once more asserted itself, as by degrees he slowly crept back to health. Three long months went by, and at last the verdict came, once more might that wee Donald hope to join in the work and play of his fellows. "A miracle," the surgeon called him, but I think the miracle, under "Le Bon Dieu," was the skill and nursing he received. One day he was put under chloroform to have a final examination, and a short time after I was passing the ward his sharp eyes saw me. "Nurse," he shouted, "I've been 'upstairs,' and ye ken, the doctor says, I'm to play the 'pipes'" (his great ambition being to play the bag-pipes in one of Her Majesty's Highland regiments). With returning health his spirits rose, and it was difficult to maintain discipline with him, for he was irrepressible, and the men, I fear, encouraged him, he was such a favourite. But I believe the influence of the wee laddie made for good with some of these rough, hard men, now, perhaps, for the first time on a bed of pain and weariness, finding time for reflection, and opportunity for turning to better things. One instance can be given, that of a man known to be a drunkard and a bad liver, who was an occupant of the same ward; his one redeeming feature was a love for children, and wee Donald soon crept into his heart. After his discharge he came again and again to the dispensary window. "I've come for a word of wee Donald," he would say; "tell him it was Bruce was 'speiring' (asking) for him; ye ken he was fond of me, and I was the only one he minded when he was bad," perhaps laying down a penny for the wee boy. A look of better things would come into his face as he said, "It's hard to keep straight, but I'm getting a job." And we remembered the words: "A little child shall lead them."

At last wee Donald was taken out of his "box," and able to wriggle about once more. Several bad accidents came in just then, and as Donald was able to be moved he was sent to the convalescent home. But how we missed our little torment; and as the men often say, "Aye! but it's not the same place since wee Donald went."

### Everybody's Opinion.

[Correspondence on all subjects is invited, but we cannot in any way be responsible for the opinions expressed by our correspondents. No communication can be entertained if the name and address of the correspondent is not given, as a guarantee of good faith but not necessarily for publication, or unless one side of the paper only is written on.]

### PREMIUM PROBATIONERS.

"A Would-be Nurse" writes: I was very pleased to read "A New Pro.'s" experience in your paper, and I also thank the writer very much for the information therein. I hope she will write again shortly; her letters are very useful to would-be nurses. I want to become a nurse myself, so you will understand why I like reading the experience of other nurses.

### MADRAS GENERAL HOSPITAL.

"A Looker-on (Madras)" writes: In your issue of July 28th I was interested to find that the General Hospital, Madras, was not too far away to have the benefit of your valuable paper. A "Resident in Madras" will have done a good work if, through your columns, the "local government" can be brought to give their attention to the management of the Madras General Hospital, the unsatisfactory state of which has for several years been a grievance to many interested in the welfare of the hospital and nursing staff. The recent "increase of pay" cannot fail to gratify the nurses who receive it, but even with the increase the difficulty of retaining suitable nurses remains. And since, to quote the words of a member of the staff who knows, "there are dozens of worthy people waiting for vacancies," the difficulty is not in obtaining them—wherein does it lie? The reason is decidedly not one of "pay."

### A PRIVATE NURSING INSTITUTE.

"Inquirer" writes: In glancing through your columns my attention was drawn to the statement of a principal leaving the important duties of a private nursing institute to an "untrained woman." Now an untrained person is confessedly an irresponsible person as regards medical matters, but ignorance may be allowed to cloak her sins of omission as regards nurses. It, therefore, seems unfair to me to call attention particularly to her faults. The case of the principal who is "absent" is very decidedly open to question, and in the interests of the honourable profession who largely support and of the community who either benefit or suffer at the hands of such nursing institutions, it is worth while inquiring if so careless a principal can be other than an "untrained" woman herself; on the other hand, if trained, is she not culpably negligent of the duties entrusted to her? Some time ago I met a nurse at a private boarding house who casually told me her institution had sent her there to isolate after scarlet fever. I was astonished at the time, but concluded that nurses' institutions, being under experienced and professional management, must know what was right—wrong though it seemed, the idea of untrained and ignorant women being in such positions never occurring to me. Perhaps, however, overflow and infectious nurses are sent from such institutions and by such people to the same houses. If so, no one need look farther for the cause of the frequent appearance of infectious maladies, where least expected and without apparent cause. Of late we have heard much of inexperienced work given in the name of charity and as a labour of love. This is at least honest from a business point of view, no fees being asked nor taken, inexperience being simply offered and accepted in time of need. This is, however, inadmissible as regards institutional work, which is universally supposed to be conducted upon the highest conscientious and the strictest of business principles. When the too trusting public—be they either time-pressed and busy doctors or anxious friends in the hour of acute sickness—apply for help to a nursing institute, they fully depend upon being met by skilled and kind attention, and by virtue of the high fees charged have every right to expect it. All are willing to leave the issues of sickness to a higher Power than that of any human aid, but I very much question if anyone cares to pay unprincipled principals for the relegating of their vital interests to either chance or the Lord.

### NURSING IN AMERICA.

"An English Nurse" writes: Owing to absence from home, I have only just read "An American Graduate's" rejoinder to my articles on "Nursing in America." I must confess to being somewhat surprised at the tone she in some instances adopts, but on reading the rejoinder carefully I see she must have read my articles in a very perfunctory manner. Surely, I praised St. Luke's Hospital in a way that should have satisfied even a graduate of that institution. Of its working or methods of nursing I expressly said I could learn nothing, as I was shown over that establishment by quite a new probationer. I find, too, that "An American graduate" quotes as my opinion and my experiences what, if she had read more carefully, she would have found were the views of American nurses themselves—women like herself, with from ten to fifteen years' experience of nursing. It was they, and not I, who sat for weeks waiting for work last winter. Even in her criticism of my allusion to the incident of the quack doctor she evidently did not take the trouble to notice that in acting as I did I simply carried out the orders of the doctor in charge of the patient. It was a matter of yielding to the feelings of relatives, as the patient was absolutely beyond hope and quite unconscious. Surely ordinary humanity would dictate such a course under the circumstances. What I said about the hospital in the mountains happens to be true in every particular, even to patients and nurses dining off the same joint, which only shows that "An American Graduate" does not know her own country. However, as it is a pretty big one, and a perfect knowledge of our native land is a sin of which we English are not guiltless, one cannot enlarge on that score. As regards the rate of remuneration, I am quite aware that in Boston and New York a nurse is usually paid from twenty to twenty-five dollars a week. At a lecture given by a nurse last winter at the Nurses' Club in Boston, she stated that fifteen dollars a week was all a nurse in San Antonio or the surrounding district could expect to get, and at that rate they are, I know, paid at places as far apart as Minneapolis and Seattle. By a printer's error in my last article three pounds a week was made into three dollars as the amount a nurse could earn up in the mountains. I am afraid from the tenour of "An American Graduate's" letter, that she has not had so good a time on this side as I had on the other, where, as I stated, I received the greatest and most generous kindness from everyone. If so, I am sorry. I have nursed in England for over sixteen years, first as hospital nurse, next as a private nurse, latterly as a superintendent (during which I came into contact with all sorts and conditions of people—surgeons and physicians of world-wide fame, country practitioners, people of the highest rank, and labouring people), and I never in any instance was treated with anything but kindness and consideration by doctors and patients alike. In conclusion, may I quote from a letter received from an American the other day: "We have read your articles as they came out with the greatest pleasure. You have grasped the situation, and have succeeded in being fair—a task not always easy. Your description of us all is both graphic and truthful."

### BARBADOS GENERAL HOSPITAL.

Mr. D. Wilson, the Secretary and Manager of Barbados General Hospital, writes us on behalf of the board of directors under date of August 22nd as follows: The attention of the board of directors has been called to an article on the above in your issue of July 21st which in the opinion of the board is calculated seriously to injure the efficiency of the institution. You say a former matron resigned because she was "not able to work with the committee." That is not strictly accurate. But suppose that had been the reason given for resigning is it an unheard-of thing for a servant to resign a situation because rules are laid down which for some reason or other they do not agree with? and with whom rests the authority to settle rules for the working of such an institution? If the matron to whom you refer could not accept the rules laid down by the committee, were they to yield in favour of the discontented matron? Again, you say, "her successor arrived about November, and it is obvious that she too could not tolerate the position." If you mean by this that the present matron has tendered her resignation because she could not "work with the committee" it is untrue. You also quote a paragraph from a Barbados paper which says, "That there should be disorganisation is only what is to be expected at an institution supported by the public and yet with an administration independent of the public and everyone else." This is arrant nonsense. And a glance at the board of administrators will show that it is as representative as a board well can be. And to any unprejudiced person its constitution is as sufficient a safeguard against mismanagement of any kind (including tyranny to matrons) as the public well can have. The following are the board of administration: His Excellency Sir James Hay, K.C.M.G., Governor of Barbados, representing the whole colony; his Lordship the Bishop of Barbados, representing the great body of Church people of the colony; the President of the Legislative Council (Sir Geo. C. Pile); the Honourable B. C. Howell, M.L.C.; the Honourable Timothy Yearwood, M.L.C.; the Honourable E. B. Colvin, M.L.C.; his Honour the Speaker of the House of Assembly; the Honourable W. Herbert Greaves, Q.C., Attorney-General, M.C.P.; the Honourable G. Aubrey Goodman, Solicitor-General, M.C.P.; Charles T. Cottle, Esq., M.C.P.; Thomas Parris, Esq., M.C.P.; H. E. L. King, Esq., M.C.P.; H. Graham Yearwood, Esq., M.C.P.; T. W. B. O'Neal, Esq., M.C.P.; H. Walter Reece, Esq., M.C.P.; H. E. Thorne, Esq., M.C.P.; Thomas Bowen, Esq., M.D.; Edwin Racker, Esq., J.P.; Joshua Baeza, Esq. The board, in noticing the remarks in your paper, are not seeking to justify their management in any way. Nor would your statements have been noticed by them had your article not appeared to injure the hospital by the warning you give to those who might wish to reply to the advertisement for nurse and matron. They are of opinion that in taking the unsupported testimony of some aggrieved informant as the basis of your warning against the whole body of directors, you have as far transgressed the limits of what is just as of what is good taste; and they regret exceedingly that your paper should have made it possible for anyone to think that the welfare of an institution of such benefit to the public should not be the first object that the board of management has in view.

*** We did not say a word about the board of administration, but if several matrons find it impossible to work with the committee the fault can hardly always lie with the matron. The lady referred to at the outset of Mr. Wilson's letter was, we are informed, the twelfth head nurse within twenty years, though the appointment is for three years.—Ed. *T.H.*

### For Reading to the Sick.

"A NEW commandment I give unto you, that you love one another as I have loved you."

Strengthen our love, O Lord, that we
May in Thine own great love believe,
And, opening all our soul to Thee,
May Thy free gift receive!
All powers of mind, all force of will,
May lie in dust when we are dead;
But love is ours, and shall be still
When earth and seas are fled.

—*T. C. Maxwell.*

Talk not of wasted affection, affection never was wasted;
If it enrich not the heart of another, its waters, returning
Back to their springs, like the rain, shall fill them full of refreshment;
That which the fountain sends forth returns again to the fountain.
Patience! accomplish thy labour! accomplish thy work of affection!
Sorrow and silence are strong, and patient endurance is God-like;
Therefore accomplish thy labour of love, till the heart is made God-like,
Purified, strengthened, perfected, and rendered more worthy of heaven.

—*Longfellow.*

**Reading.**

We cannot love our Father without going on to love our fellows. "If a man say, I love God, and hateth his brother, he is a liar? for he that loveth not his brother whom he hath seen, cannot love God whom he hath not seen. And this commandment have we from Him, that he who loveth God love his brother also." "As long as any one has the means of doing good to his neighbours, and does not do so, he shall be reckoned a stranger to the love of the Lord."—*V. Staley.*

The practical Christian, then, will regulate his conduct towards his fellows, high and low, rich and poor, by the golden rule of brotherly love. He will steadily refuse to isolate himself as a solitary unit, an only son, from those who stand in the same relation to God as himself. He will enlarge his heart to take in the interests of others, and be careful to respect their rights. He will, in short, strive to "fulfil the royal law, according to the Scripture, Thou shalt love thy neighbour as thyself." And he will do all this on principle, for a definite reason, because he believes in the Fatherhood of God.—*Ibid.*

Love demands a return of love. And the highest love seeks the highest object of love, in the return of love from that object. Man's love for nature and his fellow men may satisfy the craving for affection up to a certain point, but only to a certain point. "We needs must love the highest when we see it," but that highest is not to be found in man, but in God. All human love is more or less imperfect, and however faithful it may be, before long a separation must come—one will be taken and the other left. Human love, beautiful as it is, can never completely satisfy the deep yearning of the human heart, with its capacity for infinite love. There remains the void which One alone can fill, and that One is the Infinite and Personal God.—*Ibid.*

Bengel, in one of his incomparable epigrams, gives the history of the soul through its relations to fear and love. "Without fear and love; with fear without love; with fear and love; with love without fear."

Since that loving Lord
Commanded us to love them for His sake,
Even for His sake, and for His sacred word,
Which in His last bequest He to us spake,
We should them love, and with their needs partake;
Knowing that, whatsoe'er to them we give,
We give to Him by Whom we all do live.—*Spenser.*

### Notes and Queries.

**THE Editor is always willing to answer in this column, without any fee, all reasonable questions, as soon as possible.**

**But the following rules must be carefully observed:—**

1. **Every communication must be accompanied by the name and address of the writer.**
2. **The question must always bear upon nursing, directly or indirectly.**

**If an answer is required by letter a fee of half-a-crown must be enclosed with the note containing the inquiry.**

*Cookery.*

(247) Could you tell me if it is possible for a girl of 21 to make her living by cookery? What is the best school, and what are the fees?—*L. S.*

The study and teaching of cookery is becoming more important every year. You could study at the National Training School of Cookery, Buckingham Palace Road, S.W. A course of training for a teacher's cookery diploma occupies 40 weeks, and costs £30. Full information on this subject is given in the Epicure Directory, Granville House, Arundel Street, W.C.

*Sanitary Inspection.*

(248) Would you kindly tell me of any public institution where I could qualify as sanitary inspector?—*Emily B.*

The Sanitary Institute, Margaret Street, W., or Bedford College, Baker Street, W. Apply for particulars to the Secretary.

*Cramp.*

(249) Can anyone tell a nurse on night duty what to do for cramp in both legs and feet? It comes on in the day time, and is sometimes so bad as to prevent her having any sleep.—*F. W.*

Firstly, when able to sit down on duty always use a leg rest (a high stool or another chair will answer the purpose, which is to take the weight off the leg muscles). Secondly, massage the feet and legs well before lying down, beginning with the toes and working upwards (you can do this for yourself). Thirdly, don't stand about where it is possible to sit down.

*Sanitary Inspector.*

(250) 1. Do you think it desirable for a trained nurse to take a post as sanitary inspector? 2. Are there many lady inspectors employed? 3. And is Mr. ——, the best teacher?—*Sister.*

1. Many trained nurses are glad to have the chance of an appointment as sanitary inspector. 2. There are not very many ladies employed in that capacity as yet, but there is a prospect that the number will increase considerably. 3. It is impossible for us to comment on the qualifications of private teachers.

*L.O.S.*

(251) I should be glad of some particulars as to the L.O.S. Is it a difficult examination, and would a certificated midwife require further instruction before going up for it? Are the examinations held at stated times?—*Nurse A.*

The L.O.S. holds examinations four times a year. Apply the Secretary, 20, Hanover Square, W., for syllabus and regulations. It is a fairly difficult examination, but a well instructed midwife ought to be able to pass it without further training.

*Foreign Training.*

(252) Will you kindly tell me if there is a private institute or nursing home in Nice or Rome where probationers are trained, or where candidates with two years' fever training are accepted?—*E. E. C.*

There is no training school (as we understand the term) for English nurses abroad. The Nice Institute (formerly the Hollond Institute), Villa Soleil, Avenue Thiers, Nice, might possibly be able to find employment for a fever nurse.

*Visiting Nurse.*

(253) Will you kindly tell me the usual fee (moderate) of a professional nurse taking daily cases, where another nurse takes night duty?—*Nurse.*

Your query does not state the length of your daily visit; it may be half an hour or all day, and the fee is always in proportion to the time occupied. For a single visit of the former time 2s. 6d. is sometimes charged; for a course of visits from 10s. to 15s. weekly.

*L.O.S.*

(254) Can you tell me where I could receive evening instruction to qualify me for L.O.S.? I have already the maternity nurse's certificate of the City of London Lying-in Hospital, and as I am working for a society I have only the evenings to spare. 2. What would the cost be?—*M. H.*

The Secretary of the Midwives' Institute would be pleased to send you information as to the courses of study provided by the Institute. The fees are very moderate. Address 12, Buckingham Street, Strand.

**Standard Books of Reference.**

"The Nursing Profession: How and Where to Train." 2s. net; post free, 2s. 4d.

"The Nurses' Dictionary of Medical Terms." 2s.

"Burdett's Series of Nursing Text-Books." 1s. each.

"A Handbook for Nurses." (Illustrated.) 5s.

"Nursing: Its Theory and Practice." New Edition. 3s. 6d.

"Helps in Sickness and to Health." Fifteenth Thousand. 5s.

"The Physiological Feeding of Infants." 1s.

"The Physiological Nursery Chart." 1s., post free 1s. 3d.

**All these are published by THE SCIENTIFIC PRESS, Ltd., and may be obtained through any bookseller or direct from the publishers, 28 & 29, Southampton Street, London, W.C.**

